# Bile acids and incretins as modulators of obesity-associated atherosclerosis

**DOI:** 10.3389/fcvm.2024.1510148

**Published:** 2025-01-06

**Authors:** Andrijana Kirsch, Juergen Gindlhuber, Diana Zabini, Elena Osto

**Affiliations:** ^1^ Division of Physiology and Pathophysiology, Otto Loewi Research Center for Vascular Biology, Immunology and Inflammation, Medical University of Graz, Graz, Austria; ^2^ Vetsuisse Faculty, University of Zurich, Zurich, Switzerland

**Keywords:** cardiovascular disease, obesity, gut hormones, lipoproteins, atherosclerosis

## Abstract

Obesity is one of the major global health concerns of the 21st century, associated with many comorbidities such as type 2 diabetes mellitus (T2DM), metabolic dysfunction-associated steatotic liver disease, and early and aggressive atherosclerotic cardiovascular disease, which is the leading cause of death worldwide. Bile acids (BAs) and incretins are gut hormones involved in digestion and absorption of fatty acids, and insulin secretion, respectively. In recent years BAs and incretins are increasingly recognized as key signaling molecules, which target multiple tissues and organs, beyond the gastro-intestinal system. Moreover, incretin-based therapy has revolutionized the treatment of T2DM and obesity. This mini review highlights the current knowledge about dysregulations in BA homeostasis in obesity with a special focus on atherosclerosis as well as athero-modulating roles of incretins and currently available incretin-based therapies.

## 1 Introduction

Obesity is a globally increasing epidemic (1, 2) associated with comorbid conditions such as type 2 diabetes mellitus (T2DM), metabolic dysfunction-associated steatotic liver disease, and atherosclerotic cardiovascular disease (CVD) (3–6). Atherosclerosis is a systemic chronic inflammatory disease characterized by endothelial dysfunction, accumulation of lipids, immune-inflammatory cells, and fibrous neointimal tissue in the arterial wall, leading to the formation of plaques (7–9). Endothelial dysfunction, which is characterized by lower bioavailability of the vasorelaxing nitric oxide (NO) and increased production of the vasoconstricting endothelin-1 (ET-1), is, together with a dysregulated metabolism of low density lipoprotein (LDL) and high density lipoprotein (HDL), a key player in atherosclerosis onset and progression (8, 10–13). LDL cholesterol has a well-established causal role in the development of atherosclerosis (14, 15). Elevated circulating levels of LDL cholesterol after deposition in the arterial intima, undergo oxidation, becoming pro-inflammatory and attracting monocytes-macrophages (16). Macrophages engulf oxidized LDLs becoming foam cells, a hallmark of early atherosclerotic lesions (17). Over time, the accumulation of foam cells, along with other cellular debris, leads to the formation of fatty streaks and progression to advanced and rupture-prone plaques (18–20). In contrast to LDL, HDL cholesterol is often termed the “good” cholesterol. This is, however, an oversimplification of the complex physiological actions of this class of lipoprotein. The best known function of HDL is to mediate reverse cholesterol transport (RCT), by which excessive cholesterol is removed from arterial walls and peripheral tissues and transported back to the liver for excretion or reuse to synthetize hormones (21, 22). While low levels of HDL-cholesterol increase the risk for CVD, elevating HDL levels by pharmacological inhibition of cholesteryl ester transfer protein (CETP), an enzyme catalyzing the transfer of cholesterol from HDL to LDL, and triglycerides from LDL to HDL, did not result in improved cardiovascular outcome (23–25). This disappointing result highlighted that the function of the diverse molecular components of HDL rather than solely its cholesterol content is crucial in reducing cardiovascular risk (26, 27). Once dysfunctional, HDL loses its protective RCT capacity and fails to prevent LDL oxidation (oxLDL), becoming pro-inflammatory and pro-atherosclerotic (28).

BAs are amphipathic molecules synthesized from cholesterol in the liver. BAs play a crucial role in the intestinal digestion and absorption of dietary fats (29). Beyond their digestive functions, BAs are important signaling molecules. Among several receptors activated by BAs, the most studied are Farnesoid X Receptor (FXR) and G protein-coupled bile acid receptor 1 (GPBAR1), also known as TGR5, which are present in most cell types and pathophysiological processes associated with atherosclerosis development (30–32). In obesity, increased BA production in the liver and slightly elevated BA levels in the systemic circulation are reported (33, 34) as well as reduced circulating concentrations (35). Furthermore, obesity-induced changes in the gut microbiome composition are associated with altered conversion rates of primary to secondary BAs, which may alter BA-mediated FXR and TGR5 signaling (36). Physiologically, TGR5 receptor activation in the intestine by BAs promotes the release of incretins, which exert vaso-protective actions (37, 38).

Incretins, glucose-dependent insulinotropic polypeptide (GIP) and glucagon like peptide 1 (GLP-1), are gut hormones that induce insulin release from the pancreas in a glucose-dependent manner (39, 40). GIP and GLP-1 act on multiple target cells via G-protein coupled receptors GIPR and GLP1R, respectively. These receptors are expressed in numerous organs including bone, heart and blood vessels (41). The incretin signaling is impaired in obesity and T2DM (42).

Current research on the pathophysiology of atherosclerosis associated with obesity is exploring the role of BAs and incretins (43, 44). This mini review summarizes current evidence on the role of BAs, incretins, and incretin-based therapies in modulating atherosclerosis.

## 2 BA, incretins and atherosclerosis

### 2.1 Bile acids

**Figure 1:**
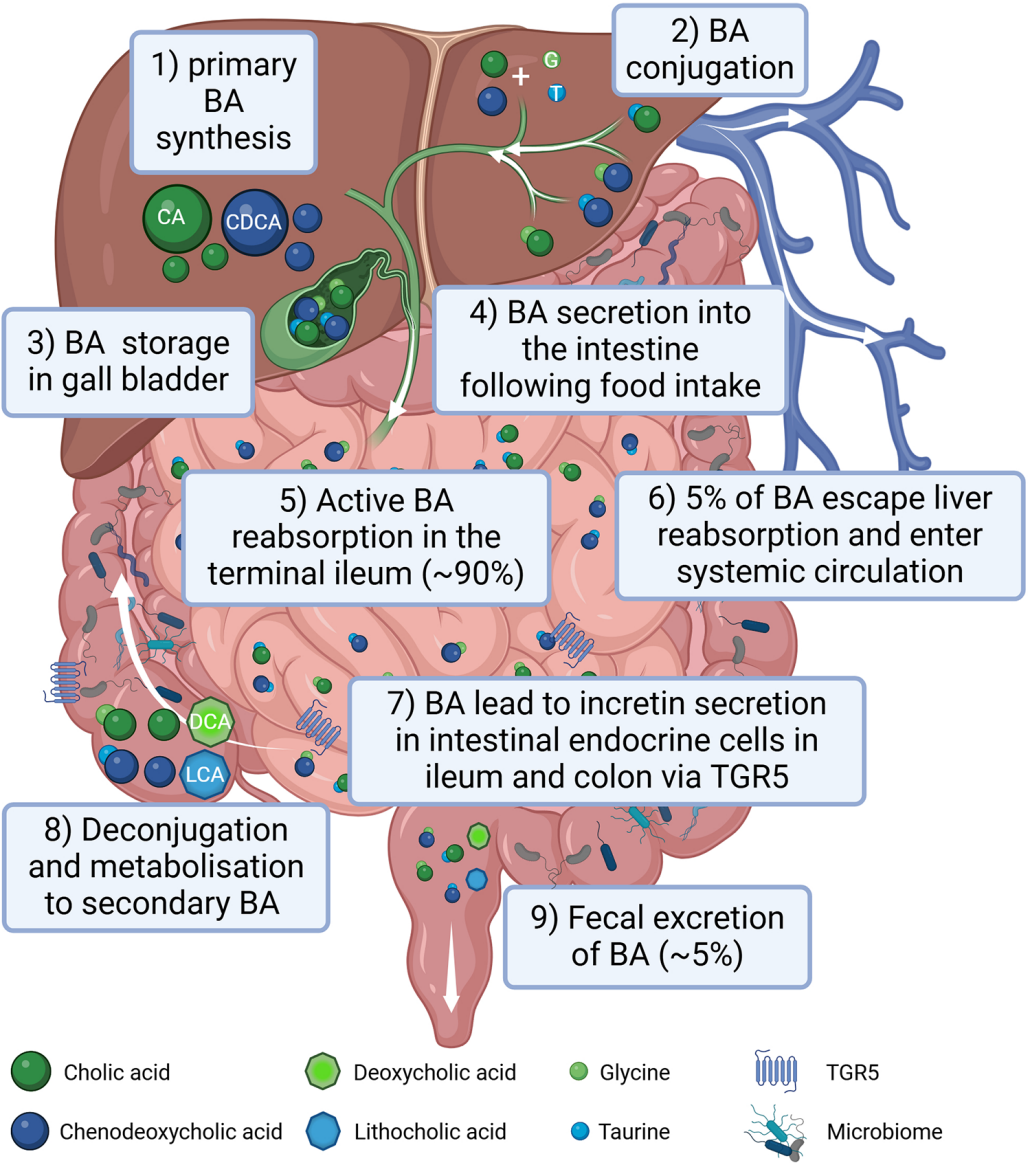
Graphical summary of bile acid (BA) metabolism in humans. BAs are synthesized in the liver (1), conjugated with amino acids, mainly glycine (G) and taurine (T) (2) and stored in the gall bladder (3). Following food intake, BAs are released into the small intestine to aid lipid absorption (4). Around 90% of the BA are reabsorbed in the terminal part of the ileum (5) reaching the liver via the portal circulation. Only around 5% of BAs escape liver reabsorption and are found in the systemic circulation (6). In the terminal ileum as well as in the colon BAs stimulate incretin secretion of intestinal endocrine cells via activating the TGR5 receptor (7). In the intestine BAs can be processed by the gut microbiota (8). Following an initial deconjugation the primary cholic acid (CA) is converted into deoxycholic acid (DCA) and chenodeoxycholic acid (CDCA) is converted into lithocholic acid (LCA). Only a small percentage of the BA is excreted with the feces (9). Created in BioRender. Gindlhuber, J. (2024) https://biorender.com/w38u902.

BAs are classified into two main types: primary BA, such as cholic acid and chenodeoxycholic acid and secondary BAs, (i.e., deoxycholic acid and lithocholic acid) (45), the latter originating by bacterial modification in the intestine (46) via deconjugation and dehydroxylation processes (47). In the post-prandial phase 90%–95% of BAs are reabsorbed in the ileum and transported back to the liver. After their almost complete reabsorption, BAs are stored in the gallbladder and await to be secreted into the duodenum upon food intake. Around 5% of total BAs escape liver reabsorption and are found in the systemic circulation reaching serum concentrations of around 1–3 µM in healthy lean individuals (48). A graphical summary of BA metabolism is provided in Figure 1.

**Figure 2:**
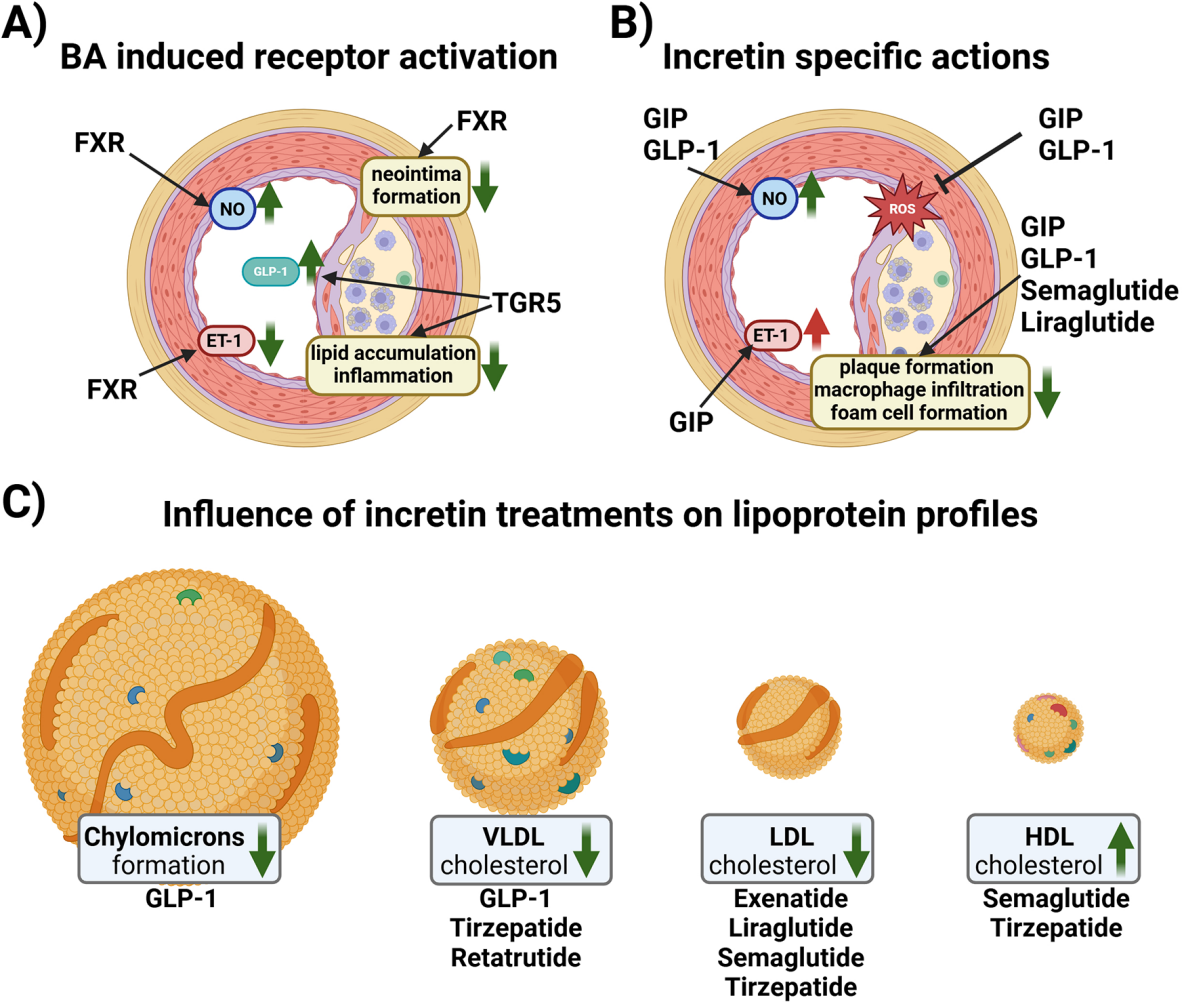
**(A)** Schematic overview of reported effects of BA receptor activation in vascular cells. In endothelial cells (ECs), Farnesoid X Receptor (FXR) activation increases nitric oxide (NO) production and reduces endothelin-1 (ET-1) expression contributing to vasorelaxation. Vascular smooth muscle cells reduce their proliferative activity upon FXR activation resulting in reduced neo intima formation. G protein-coupled bile acid receptor 1 (TGR-5) activation in endocrine cells increases the amount of glucagon-like peptide 1 (GLP-1) in the systemic circulation. Macrophages react to TGR5 activation with reduced lipid uptake and a reduction in inflammatory signaling. **(B)** Schematic overview of the effects of incretins and incretin-based therapy on ECs and plaque formation. GLP-1 induces NO production, while glucose-dependent insulinotropic polypeptide (GIP) induces the production of both NO and ET-1. Both GIP and GLP1 reduce the formation of reactive oxygen species (ROS). Native GIP and GLP-1, as well as GLP1Ras, semaglutide and liraglutide, reduce plaque formation, macrophage infiltration and foam cell formation. **(C)** Native GLP-1 decreases chylomicron formation and VLDL cholesterol levels, while GLP1RA and GLP1R/GIPR co-agonist therapy ameliorates the lipoprotein profile of patients by lowering VLDL and LDL cholesterol, and increasing HDL cholesterol. Created in BioRender. Kirsch, A. (2024) https://BioRender.com/u26n602.

BAs-dependent activation of FXR in human liver cell lines, upregulates LDL receptor expression and activity and inhibits its degradation leading to a reduction of LDL cholesterol levels (49–51). However, FXR also reduced the main HDL apolipoprotein ApoA-I transcription, decreasing HDL levels in murine animal models (52). FXR and LDL receptor double knockout male mice were protected from atherosclerosis contrary to female double knockouts (53). FXR and apolipoprotein (Apo) E double knockouts showed severe plaque formation compared to wild-type, and single FXR-/-, and ApoE-/- mice (54). *In vivo* studies in rats have shown that FXR activation is beneficial in different vascular cell types [e.g., endothelial cells (ECs) and vascular smooth muscle cells] to revert their pro-constrictory and pro-inflammatory phenotype (Figure 2A), as well as neo-intima formation, all changes, which are promoting atherosclerosis development (55–58). FXR activation enhances NO production and reduces ET-1 expression contributing to vasodilation in isolated rat pulmonary ECs (59, 60). On the other hand, hepatic TGR5 stimulation prevents hepatic BA and ectopic lipid accumulation in different murine models (61–63). Activation of TGR5 in macrophages is beneficial because it attenuates foam cell formation and inhibits the activation of inflammation, as evidenced by genetically modifying TGR5 in murine peritoneal macrophages (64). An increase in specific BA subspecies has been associated with atherosclerosis in human and animal studies. For instance, in T2DM patients carotid intima media thickness (cITM), a surrogate marker of subclinical atherosclerosis, was associated with higher deoxycholic acid and taurodeoxycholic acid levels, and lower levels of taurocholic acid than patients with normal cITM (65, 66). Conversely, the glycine conjugates of cholic acid and deoxycholic as well as lithocholic acid were found to be protective when atherosclerosis patients were compared to a control cohort (67). Of note, a disrupted BA signaling impairs glucose and lipid metabolism, exacerbating conditions like insulin resistance and fatty liver disease, which are major pro-atherosclerotic metabolic derangements (68, 69).

### 2.2 Incretins

GIP is secreted by the duodenal and jejunal K cells upon ingestion of carbohydrates and lipids, while GLP-1 is secreted by the ileal L cells (70, 71). Along with the induction of insulin secretion, GIP and GLP-1 reduce gastric emptying, and GLP-1 lowers glucagon secretion (71, 72). Physiologically, these hormones have a half-life of just a few minutes upon secretion, as GIP and GLP-1 are rapidly cleaved and inactivated by diaminopeptidyl peptidase-4 (DPP4) (73).

### 2.3 GIP

*In vitro* studies in ECs have shown that GIP have both anti- and pro-atherogenic effects. In human umbilical vein ECs (HUVEC) and canine portal vein EC, GIP induced NO production (74, 75) and reduced advanced glycation end products-induced oxidative stress and inflammation (76) but was also reported to increase ET-1 (74, 77, 78), (Figure 2B).

Monocyte-macrophages transformation into foam cells contributes to the pathogenesis of atherosclerosis (79–81). GIPR is expressed in human monocytes, mouse peritoneal macrophages and human monocyte-derived macrophages, with the GIPR expression in human monocytes being higher than in the differentiated macrophages, at least *in vitro* (82). Moreover, GIP exerts anti-inflammatory effects by suppressing lipopolysaccharide-induced tumor necrosis factor-α (TNFα) or inducible NO synthase (iNOS) in human monocyte THP-1 cells (83), as well as suppressing the chemokine ligand 2 (CCL2)-induced migration also in mouse monocytes (84).

Animal studies using ApoE-/- deficient mice show anti-atherogenic effects of GIP. The infusion of active GIP (25 nmol/kg/day) for 4 weeks blunted the aortic plaque formation and macrophage accumulation within the plaque (82). Moreover, decreased foam cell formation and downregulation of the scavenger receptor CD36 and cholesteryl ester-forming acyl-coenzyme A: cholesterol acyltransferase-1 in macrophages was reported (82). Anti-atherogenic effects were also observed in streptozotocin-induced diabetic ApoE-/- mice, where GIP infusion led to a reduction of aortic plaque formation, intra-plaque macrophage accumulation and macrophage foam cell formation (85). Moreover, overexpression of GIP has been reported to stabilize the atherosclerotic plaque in non-diabetic ApoE-/- mice by blocking monocyte/macrophage activation (84). The anti-atherogenic effect of GIPR- agonism has been described also in LDLr -/- mice fed with a high fat, high cholesterol diet. Treatment of these mice with a long-acting acylated GIP analog reduced dyslipidemia and atherosclerotic plaque formation (86). Loss of GIPR induced aortic atherosclerosis and inflammation in ApoE−/−:Gipr−/− high fat diet-fed mice despite a reduced weight gain and preserved glucose homeostasis compared to ApoE−/−:Gipr+/+ mice (87), further confirming the anti- inflammatory role of GIP in atherosclerosis (Figure 2B).

### 2.4 GLP-1

Native GLP-1 has been shown to be atheroprotective *in vitro* as it stimulates the production of vasodilatory NO in ECs (35, 75). Similar to GIPR, GLP1R is also expressed in macrophages, and treatment with native GLP-1 decreased the uptake of oxLDL and expression of CD36 in human monocyte-derived macrophages (88). Administration of active GLP-1 to ApoE -/- mice significantly suppressed atherosclerotic lesions and macrophage infiltration in the aortic wall compared to vehicle controls (82). Infusions of recombinant GLP-1 in rats dramatically decreases intestinal lymph flow and reduces triglyceride absorption and ApoB and ApoA-IV production (89). Moreover, portal vein injections of GLP-1 in hamsters and mice decreases postprandial chylomicron (CM) and VLDL secretion via vagal afferent nerves originating in the portal vein (90). These GLP-1 effects could contribute to its atheroprotection, as remnant CM and VLDL have atherogenic properties (91) (Figure 2C).

## 3 Incretin-based therapy and modulation of atherosclerosis

Several classes of incretin-based drugs have been developed to treat T2DM, including DPP4-inhibitors and GLP1R agonists (GLP1RAs). DPP4-inhibitors will not be discussed in detail in this mini review; for an overview on the atheroprotective role of DPP-4 inhibitors in both human and animal models see (92, 93). Incretin-based drugs, especially GLP1R agonists, beyond improving glucose levels, have shown beneficial effects on the lipid profile (Figure 2C), weight reduction, and cardiovascular protective effects (94). The most commonly reported side effects are delayed gastric emptying, bloating, diarrhea and vomiting, although drug titration mitigates the incidence of these side effects (94). GLP1RA and the dual GIPR and GLP1R agonist, tirzepatide, are currently also used for weight management of overweight/obese patients with and without CVD (95).

### 3.1 GLP1R agonists

GLP1RAs activate the GLP1R and are resistant to inactivation by DPP-4 (96). The first GLP1R agonist in clinical use was exenatide (exendin-4) (97), subsequently, various GLP1RAs were developed based on the human GLP-1 peptide, including liraglutide, dulaglutide and semaglutide, which have different characteristics pertaining to route and frequency of administration, and pharmacokinetics (98).

#### 3.1.1 Preclinical studies

Mechanistic studies have addressed the effect of GLP1RAs on atherosclerosis in rodent models. GLP-1 peptide analogues CNTO3649 and exendin-4 reduced VLDL production and hepatic steatosis after 4 weeks of treatment in high fat diet-fed APOE*3-Leiden transgenic mice, a mouse model with human-like lipoprotein metabolism (i.e., high triglycerides, LDL and VLDL, low HDL) and accelerated atherosclerosis development (99). Semaglutide and liraglutide reduced atherosclerotic plaque formation in aortas of ApoE -/- and LDLr -/- mice, and semaglutide blunted gene expression of pro-inflammatory and osteogenic proteins, such as TNFα and osteopontin (100). Liraglutide alone inhibited the progression of early onset, low-burden atherosclerotic disease (101) as well as attenuated pre-established atherosclerosis in ApoE -/- mice by reducing proinflammatory immune cells and mediators (102), suppressing foam cell formation (103) and lowering the endothelial expression of the proinflammatory vascular cell adhesion molecule 1 (104).

#### 3.1.2 Clinical trials

Several randomized cardiovascular outcome trials have been conducted, showing positive effects of GLP1RA on cardiovascular risk reduction (105–111). In addition to enhancing insulin secretion, GLP1RAs may reduce postprandial chylomicron overproduction in T2DM patients by reducing intestinal absorption of dietary lipids and enhancing hepatic fatty acid oxidation (112). Exenatide and liraglutide have been reported to be equally effective in lowering postprandial dyslipidaemia, an effect observed immediately after initial administration, as well as after a two-week treatment period (113). In a double-blind, randomized, placebo-controlled, crossover study with subjects who exhibited impaired glucose tolerance or had recent-onset T2DM, a single subcutaneous injection of exenatide strongly and consistently inhibited the postprandial increase of proatherogenic lipids and lipoproteins (114). A clinical study in patients with T2DM treatment with a long-lasting release exenatide on top of metformin, a first-line therapy for T2DM, led to improved cardiometabolic parameters, including cITM and flow-mediated dilation (115). In two prospective studies, liraglutide treatment decreased cITM, total- and LDL-cholesterol as well as triglycerides after 8 months of treatment in T2DM patients, as well as during an 18-month follow-up in subjects with T2DM and metabolic syndrome (116), thereby improving cardiometabolic risk factors. Moreover, liraglutide reduced the level of atherogenic small dense LDL-3 subfraction in association with a lower cITM (117). Semaglutide also reduced cITM (118), and improved the cholesterol profile, triglyceride levels (119, 120) and reduced oxLDL (121) in T2DM patients. Further studies are needed to assess the effect of GLP1RA on other atherogenic lipoproteins such as lipoprotein(a) or electronegative LDL.

### 3.2 Dual GIPR/GLP1R agonism

Tirzepatide is the first unimolecular dual GIPR/GLP1R agonist for the treatment of T2DM and overweight/obesity (122). The co-agonism of GLP-1 and GIP results in significantly greater blood glucose and weight reduction than for GLP1R agonism alone (123, 124). Moreover, tirzepatide treatment in patients with obesity and prediabetes resulted in a lower risk of progression to T2DM compared to placebo (125). The mechanism behind the greater body weight reduction in humans is still being investigated (126).

#### 3.2.1 Preclinical studies

Animal studies suggest that GIP suppresses food intake via neural GIPR activation, although it is still not clear especially for the peripheral actions whether or not continuous GIPR agonism causes functional antagonism of the GIPR (126).

To the best of our knowledge, there are no published studies regarding the mechanism of lipid lowering effect by tirzepatide in humans. However, a recent study in APOE*3-Leiden. CETP mice, a transgenic mouse model with accelerated atherosclerosis, showed that combined GIPR/GLP1R agonism attenuated the development of severe atherosclerotic lesions (127, 128). GIPR/GLP1R agonism decreased markers of low-grade inflammation and lowered plasma triglyceride levels by increasing VLDL-derived fatty acid uptake by adipose tissue, as wells as increasing the liver uptake of VLDL remnants. In comparison, treatments with single agonists showed non-significant improvements.

#### 3.2.2 Clinical trials

SURPASS trials in T2DM patients showed that tirzepatide was superior compared to placebo and insulin glargine in lowering triglycerides, LDL-, and VLDL- cholesterol levels (129) as well as increasing HDL-cholesterol (130, 131). When compared to semaglutide or insulin degludec, tirzepatide significantly reduced VLDL cholesterol and increased HDL cholesterol, while total cholesterol and LDL cholesterol did not differ among treatments (124, 132). Similarly, in clinical trials with the focus on obesity treatment (SURMOUNT trials), tirzepatide was superior compared to placebo in lowering triglycerides, total-, LDL-, and VLDL- cholesterol levels as well as increasing HDL cholesterol (95, 133–135).

The lipid lowering effect of tirzepatide would be expected to have benefits in reducing clinical outcomes from atherosclerotic and non-atherosclerotic CVD. The recently concluded SUMMIT trial showed that tirzepatide lowered the risk of a composite death from cardiovascular causes or worsening heart failure than placebo in patients with heart failure with preserved ejection fraction and obesity (136). Other ongoing clinical trials are exploring potential cardiovascular benefits of tirzepatide in diabetic and overweight/obese participants with established CVD or high cardiovascular risk (137–139), as well as the effect of tirzepatide on the progression of coronary atherosclerosis (140).

### 3.3 Future incretin-based therapies

Tirzepatide's superiority over its mono-agonist equivalents has triggered the development of additional multi-agonistic medications as the next generation of therapeutics for metabolic disease (94).

One promising medication is retatrutide, a triple GIP/GLP-1/glucagon receptor agonist. The treatment of obese adults with retatrutide resulted in a mean weight reduction of 24.2% after 48 weeks, and was associated with improvements in cardiometabolic measures (exploratory endpoints) including systolic and diastolic blood pressure, levels of glycated hemoglobin, fasting glucose, insulin, and lipids (141). Triglycerides, total cholesterol, LDL- and VLDL-cholesterol were lower in retatrutide groups, but no improvements in HDL cholesterol levels were observed compared to placebo. In a study in T2DM patients with a BMI 25-50 kg/m^2^ retatrutide treatment significantly decreased body weight from baseline compared to placebo and dulaglutide and lowered the fasting lipid profile in a dose-dependent manner at 36 weeks (142). Higher concentrations of retatrutide (8 mg and 12 mg) significantly decreased total cholesterol, triglycerides and non-HDL cholesterol compared to placebo or dulaglutide. The non-HDL cholesterol effect was driven by reductions in VLDL cholesterol concentrations, while changes in LDL- and HDL cholesterol were generally not significantly different vs. placebo or dulaglutide.

## 4 Outlook and conclusion

BAs act as vital metabolic regulators, rather than mere digestive aids. BAs are used in traditional Chinese medicine as anti-oxidant to treat multiple digestive and metabolic disorders and in western medicine semi-synthetic BAs like obeticholic acid are treatments for cholestatic liver diseases (143–146). BAs are commercially available as dietary aids and their assumption may lead to shift in the circulating BA pool, however, since absolute serum levels are tightly regulated long-lasting BA modulation and their effect need to be further investigated (147). By activating FXR and TGR5 as well as influencing GLP-1 secretion, BAs contribute to both energy balance and cardiovascular health and future research is examining their role in obesity-associated cardiometabolic derangements. Incretins and incretin-based therapies have a multifaceted, beneficial influence on the cardiovascular function by improving EC function, reducing inflammation, pro-atherogenic lipid and progression of atherosclerotic plaques. GIP actions have recently sparked interest based on the cardiometabolic benefits of the dual GIPR/GLP1R co-agonist tirzepatide and intense ongoing research is examining how GIP co-agonism further improves the effects of single GLP1RAs in humans. Despite the clinical efficacy of incretin-based therapies, suboptimal access, high cost, limited insurance coverage and therapeutic inertia are significant barriers to their widespread adoption. Real world data regarding the long-term effect of these drugs need to be collected to fully evaluate their multi-organ mechanism(s) of action and safety.
